# A critical role for miR-184 in the fate determination of oligodendrocytes

**DOI:** 10.1186/s13287-019-1208-y

**Published:** 2019-03-29

**Authors:** Negin Afrang, Rezvan Tavakoli, Nooshin Tasharrofi, Amir Alian, Alireza Naderi Sohi, Mahboubeh Kabiri, Mehrnoosh Fathi-Roudsari, Mina Soufizomorrod, Farzad Rajaei, Masoud Soleimani, Fatemeh Kouhkan

**Affiliations:** 1 Stem Cell Technology Research Center, P.O. Box: 15856-36473, Tehran, Iran; 2 School of Paramedical Sciences, Qazvin University of Medical Sciences, Qazvin, Iran; 3 Faculty of Pharmacy, Lorestan University of Medical Sciences, Khorramabad, Iran; 4 Department of Chemistry, Rice University, Houston, TX 77054 USA; 5 Department of Biotechnology, College of Science, University of Tehran, Tehran, Iran; 6 National Institute for Genetic Engineering and Biotechnology (NIGEB), Tehran, Iran; 7 Tissue Engineering and Applied Cell Sciences Division, Department of Hematology, Faculty of Medical Sciences, Tarbiat Modares University, Tehran, Iran; 8 Department of Hematology, Faculty of Medical Sciences, Tarbiat Modares University, P.O. Box: 14115-331, Tehran, Iran

**Keywords:** NPCs, Oligodendrocytes, miR-184, SOX1, BCL2L1, LINGO1

## Abstract

**Background:**

New insights on cellular and molecular aspects of both oligodendrocyte (OL) differentiation and myelin synthesis pathways are potential avenues for developing a cell-based therapy for demyelinating disorders comprising multiple sclerosis. MicroRNAs (miRNA) have broad implications in all aspects of cell biology including OL differentiation. MiR-184 has been identified as one of the most highly enriched miRNAs in oligodendrocyte progenitor cells (OPCs). However, the exact molecular mechanism of miR-184 in OL differentiation is yet to be elucidated.

**Methods and results:**

Based on immunochemistry assays, qRT-PCR, and western blotting findings, we hypothesized that overexpression of miR-184 in either neural progenitor cells (NPCs) or embryonic mouse cortex stimulated the differentiation of OL lineage efficiently through regulating crucial developmental genes. Luciferase assays demonstrated that miR-184 directly represses positive regulators of neural and astrocyte differentiation, i.e., SOX1 and BCL2L1, respectively, including the negative regulator of myelination, LINGO1. Moreover, blocking the function of miR-184 reduced the number of committed cells to an OL lineage.

**Conclusions:**

Our data highlighted that miR-184 could promote OL differentiation even in the absence of exogenous growth factors and propose a novel strategy to improve the efficacy of OL differentiation, with potential applications in cell therapy for neurodegenerative diseases.

**Electronic supplementary material:**

The online version of this article (10.1186/s13287-019-1208-y) contains supplementary material, which is available to authorized users.

## Background

The nervous system is the information processing and central control unit in vertebrates that propagates neuronal signals to different body parts by conducting action potential along axons [1, 2]. In order to speed up the information conduction, axons are ensheathed and insulated with multi-spiral myelin membranes synthesized by oligodendrocytes (OLs) [3–5]. During neocortical development in Homo sapiens, early neural progenitor cell (NPC) differentiation into neuronal cell types, through the so-called “neurogenic phase” is temporally followed by the “gliogenic phase” during which multipotent NPCs differentiate into different glial cell types such as oligodendrocyte precursor cells (OPCs) [6, 7]. OPCs migrate to developing white matter and divide a limited number of times until reaching their target axon [8]. At the final resting sites, OPCs exit the cell cycle, turn to non-migratory phenotype, and finally differentiate into myelin-forming OLs. OLs become mainly responsible cells for myelinating adjacent axons [9]. Prevention or disturbance of this myelination process results in serious axonal damage and subsequent neuronal cell death commonly seen in severe neurological diseases such as multiple sclerosis (MS) [10–12].

To date, there is no certain cure for this disease, and the majority of approved therapy such as glatiramer acetate, interferon-beta (IFN-β), and mitoxantrone mainly target the immunological aspects of MS [13]. Thus, it is of high interest among researchers to develop a cure for MS that fights the disease by repairing the tissues and retrieving the disease. A smart approach, referred to as cell therapy, has been recently introduced that uses stem cells from the patient and differentiates them to oligodendrocyte precursor cells (OPCs) in order to regenerate the damaged tissues. This method has been employed by Thiruvalluvan et al., among others, and promising results have been achieved [14–16]. However, an efficient strategy for OL production from NPCs has not yet been devised, emphasizing the substantial need for a deeper understanding of the molecular mechanisms and epigenetic signals underlying the fate acquisition of mature OLs from pertinent progenitors.

miRNAs belong to the group of small non-coding single-stranded RNAs with 19–25-nucleotide length which through base pairing with their complementary target mRNAs accomplish their aim of gene silencing [17, 18]. There are already several reports on the role of microRNAs in inducing stem cell differentiation [19]. For instance, miR-219 and miR-338 have been identified as oligodendrocyte-specific miRNAs in the spinal cord. Overexpression of these miRNAs is sufficient to promote normal OPCs to differentiate into oligodendrocytes, both in vitro and in vivo [20]. miR-7a has also been implicated as another highly enriched miRNA in OPCs, overexpression of which in neural progenitor cells (NPCs) induces the generation of OL lineage cells [21]. Letzen et al. analyzed miRNA profiles of eight stages of OL differentiation of embryonic stem cells and reported miR-199a, miR-145, miR-214, miR-184, and miR-1183 as key differentially expressed miRNAs throughout all stages of OL differentiation [22]. miR-184, on the other hand, has been reported to show a sharply increased expression during the glial-restricted precursor (GP) to oligodendrocyte precursor (OP) differentiation stage and is also listed among top upregulated miRNAs at the final transition stages to OLs. Thus, we speculated that miR-184 might have a pivotal role in OL differentiation and normal oligodendritic development. Considering the reported roles of microRNAs in cell differentiation, which can be of high importance for developing a novel treatment for MS, and given the fact that to the best of our knowledge, there are currently no reports on the potential of miR-184 to induce OL lineage differentiation from OP, in this study, we aimed to harness their regulatory potential for better directing OL lineage specification from OP. We evaluated the impact of miR-184 overexpression in modulating differentiation pace and efficiency of NPCs both in vitro and in vivo during the development of embryonic mouse cortex towards OPCs. We also report here for the first time that miR-184 can induce OL differentiation through directly targeting a number of genes such as sex-determining region Y (SRY)-Box 1 (SOX1), BCL2 Like 1 (BCL2L1), and leucine-rich repeat and immunoglobulin domain-containing Nogo receptor-interacting protein-1 (LINGO1), which have been previously shown to be highly expressed in neurons and astrocytes and involved in inhibiting OPC differentiation.

Our findings allow us to propose an efficient approach to enhancing OL differentiation through recruiting miRNAs. This work further suggests the use of miRNAs to switch the neuronal and astrocyte-specific key genes as a valuable means for inducing a highly efficient differentiation of OLs.

## Materials and methods

### Cell lines and culture

A human NPC line, established from human-induced pluripotent stem cells (hiPSCs), was obtained from Royan Institute, Tehran, Iran [23], and used. NPCs were passaged in a 1:3 ratio for expansion on poly-d-lysine (PDL)-coated plates and cultured in neurobasal medium (Gibco) supplemented with 1× penicillin/streptomycin, 25 ng/ml bFGF, 20 ng/ml epidermal growth factor (EGF), and 2 mM L-glutamine (all from Invitrogen).

At about 70% confluency, OPC differentiation was induced according to a previously published protocol with minor modifications [24]. Briefly, NPCs were grown for 3 weeks in the oligo medium containing serum-free DMEM/HAMS F12 medium (Gibco) supplemented with 1% bovine serum albumin, 2 mM L-glutamine, 50 μg/ml gentamicin, 1× N2 supplement, 3 nM T3 (SIGMA), 2 ng/mL Shh (SIGMA), 2 ng/mL NT-3 (SIGMA), 20 ng/mL bFGF, and 10 ng/mL PDGF-AA (SIGMA). Differentiation of OPCs to OLs was initiated by growth factors withdrawn for 2 days.

Human embryonic kidney cells (HEK293T) were cultured in Dulbecco’s modified Eagle’s medium (DMEM) supplemented with 10% fetal bovine serum (FBS, Hyclone, USA) and 1% antibiotics (100 U/ml penicillin and 100 mg/ml streptomycin sulfate). Cells were grown in a humidified atmosphere containing 5% CO^2^ at 37 °C.

### Lentivirus vector construction and infection

The pLenti-III-miR-184 and miR-184 mimics were purchased from ABM. For miR-Off-184, shRNA structure of a miR-184 mutant was cloned into the pLenti-III-GFP plasmid. Empty vector (pLenti-III-Ctrl) and three different vectors with scrambled sequences (pLenti-III-Scr) were serving as controls in all experiments. Different scrambled sequences of miR-184 were designed using “GenScript” and “InvivoGene” websites and cloned into the pLenti-III vector in shRNA formats. Scrambled sequences are (I) GGAAGTGCAAGCGTGTGAAAGT, (II) ATAGGTAGTTGACGGCGGAAGA, and (III) GGACAATAGGCGTGAGTGATGA.

The packaging of the miR-184 constructs in lentiviral particles was performed by transient calcium phosphate cotransfection of HEK293T cells with 10 μg of pLenti-III-miR-184/pLenti-III-miR-Off-184 (or pLenti backbone) and 10 and 5 μg of pPAX2 and pMDG plasmids respectively. Lentiviral supernatants were harvested every 12 h for 3 days and concentrated using ultracentrifuge at 25,000 rpm for 2.5 h at 4 °C. Lentivirus titer was determined by flow cytometry analysis of GFP-positive HEK293T cells.

### Luciferase reporter experiments

3′-UTR sequences harboring potential miR-184 binding sites on the predicted target genes (SOX1, LINGO1, and BCL2L1) were cloned into the downstream of Renilla gene in the pSICHECK2 vector (Promega) between the XhoI and NotI sites. 3′-UTR cloning primers are listed in Additional file 1: Table S1. For Mut-miR-184 construction, the seeding sequence of miR-184 was changed from “GGACGGA” to “GCACTGA” and cloned into the pCDH-GFP vector. Luciferase activity was measured 48 h after cotransfection of wild-type and/or mutant miR-184 along with each target-pSICHECK2 vectors into the HEK293T cells using the dual-luciferase reporter assay system (Promega). Renilla luciferase signal was normalized to that of the Firefly as a control for transfection efficiency calculation.

### RNA extraction, RT-PCR, and quantitative RT-PCR assays

Total RNA was extracted from tissues and/or cell lines using TRizol reagent according to the manufacturer’s instructions (Invitrogen). RNA was reversely transcribed to cDNA using M-MuLV reverse transcriptase (Promega) and random hexamers (for mRNAs) or stem-loop RT-specific primers (for miR-184 and SNORD47).

Quantitative real-time PCR was performed using the ABI 157 PRISM 7500 real-time PCR system (Applied Biosystems). Normalization was performed using HPRT and SNORD47 for mRNAs and miR-184 genes, respectively. Finally, data analysis was performed using 2^−∆∆CT^ Ct method. Primer sequences are listed in Additional file 2: Table S2.

### Immunostaining and western blot assay

Immunostainings were performed 4 days after transfection/transduction against OLIG-2 and NKX2.2 (using mouse anti-OLIG-2 and mouse anti-NKX2.2 primary antibodies, from Abcam) and 2 days after the removal of growth factors against MBP on NPCs (using mouse anti-MBP primary antibody, myelin basic protein, from Abcam) followed by the secondary antibody treatments (Millipore, Billerica, MA). In each experiment, 100 cells were counted and the number of marker-positive cells was then expressed as percentages.

For western blot analysis, total protein content was extracted at 1, 2, and 3 weeks after the transduction of NPCs and blotted using standard procedures against OLIG-2, NK2 homeobox 2 (NKX2.2), myelin basic protein (MBP), BCL2L1, SOX1, and LINGO1. Horseradish peroxidase-conjugated anti-mouse antibody was used for the signal detection. Signals were developed using chemiluminescence using the ECL kit (Pierce, Rockford, IL), according to the manufacturer’s instruction.

### In utero electroporation

For in utero electroporation, pLenti-III-miR-184 or pLenti-III-Scr constructs (1 μg) in phosphate buffer saline containing 0.01% fast green were injected into the lateral ventricle of C57 mouse embryos at E14.5. After injection, electroporation was performed using five 50 ms square 40-V pulses with 950-ms intervals. Three days after electroporation, five embryos from three mice (electroporated for each construct) were collected and prepared for immunohistology and analysis [20, 21].

### Luxol fast blue staining

Luxol fast blue was utilized to stain myelin. The paraffin sections were deparaffinized and hydrated using xylene and 95% ethanol. Sections were then soaked in 0.1% Luxol Fast Blue for overnight and subsequently rinsed with 95% ethanol and distilled water to remove the excess of the stain. The slides were then differentiated by stepwisely floating in a lithium carbonate solution and 70% ethanol, each for 30 s. After the completion of the differentiation, sections were counterstained in a crystal violet solution for 30–40 s. The areas depleting Luxol fast blue were quantitatively analyzed using Leica IM 1000 image analysis software.

### Statistical analysis

Data are presented as means ± standard deviation (SD) from at least three different measurements. Student’s *t* test was used in two comparisons and values with *P* < 0.05 were considered statistically significant.

## Results

### miR-184 expanded OLIG2+/NKX2.2+ OPC populations of NPCs

**Fig. 1:**
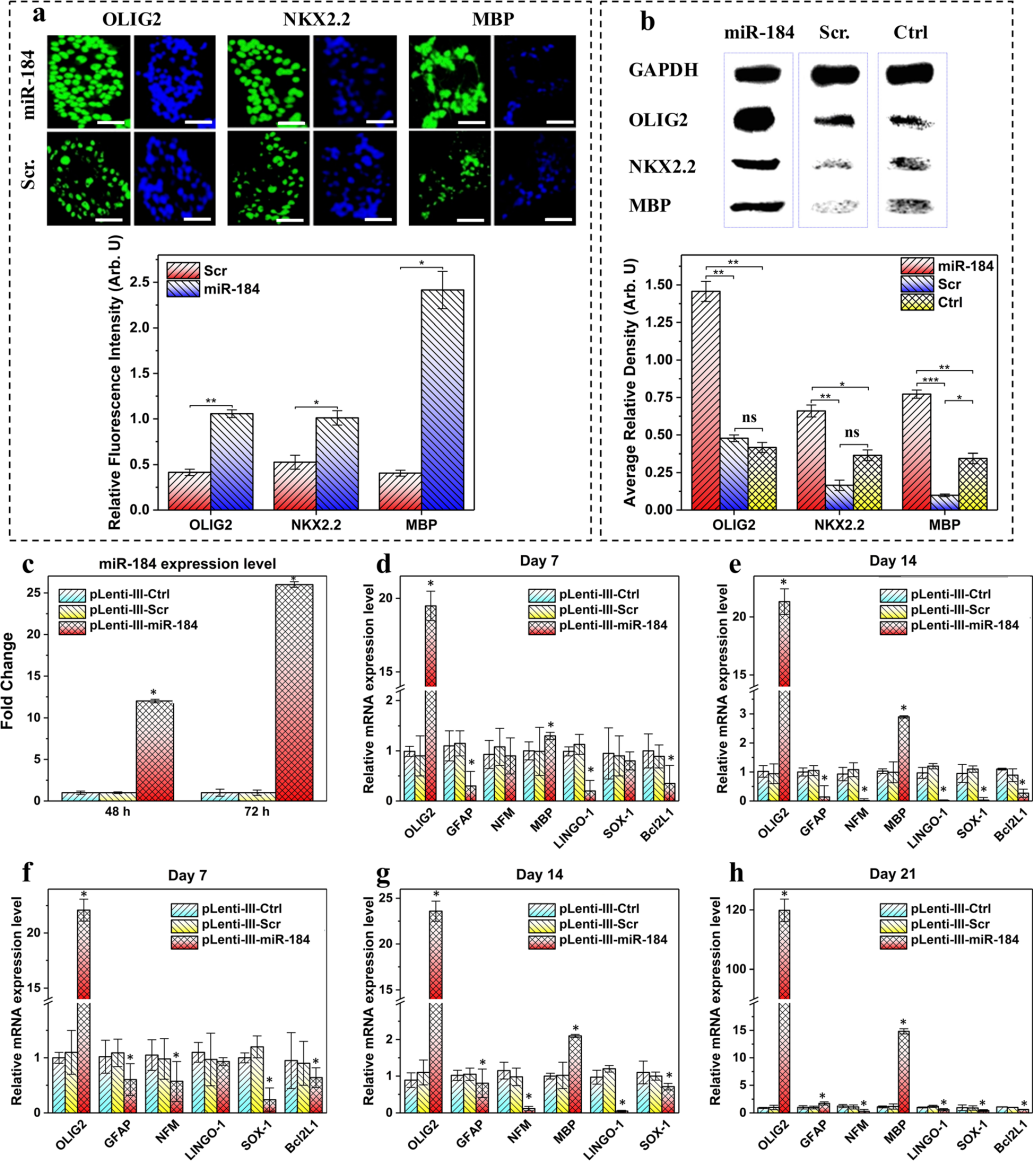
miR-184 induces OPC differentiation of NPCs. **a** The NPCs were cultured for 4 days in the oligo medium and then for 2 days in the growth factors free oligo medium. Top: NPCs were harvested and stained with antibodies against early- and late-stage OL markers, respectively. Scale bars, 50 μm. Bottom: Relative fluorescence intensity (G/B) representing the green fluorescence intensity (G) normalized to that of the blue one (B) was obtained after analysis of the images by ImageJ software. Unpaired *t*-test method was employed in each case to compare the amounts of results statistically. **b** Top: Expression of early- and late-stage OL markers analyzed by western blot. GAPDH was used as the control for normalization of protein bonds. Bottom: Average relative density of protein bands was obtained after densitometric analysis of the bands by ImageJ software followed by normalization to that of GAPDH as the internal loading control. One-way ANOVA method was used in each case to compare the amounts of results statistically. **c** miR-184 expression level evaluated by qRT-PCR in NPCs, 2 and 3 days after transduction with pLenti-III-Ctrl, pLenti-III-Scr, or pLenti-III-miR-184 relative to SNORD47 as an endogenous control. **d**, **e** qRT-PCR of lineage-specific genes from pLenti-III-Ctrl, pLenti-III-Scr, or pLenti-III-miR-184 transduced NPCs in the absence of growth factors at days 7 and 14. β-actin was used as internal control. **f**–**h** Relative expression levels of lineage-specific genes analyzed by qRT-PCR from pLenti-III-Ctrl, pLenti-III-Scr, or pLenti-III-miR-184-transduced NPCs at days 7, 14, and 21 in the presence of growth factors. β-actin was used as internal control. In the case of pLenti-III-Scr, transduction was performed with three pLenti-III-Scr construct and the values shown are the average obtained from them. Data represent mean ± SEM. Columns, mean of three replicates; bars, SD *P value < 0.05, **P value < 0.01, ***P value < 0.001. ns: non-significant (*P* value > 0.05)

Previous studies have reported miR-184 to be one of the miRNAs that are highly expressed during OL differentiation of neural progenitors [22]. To scrutinize the role of miR-184 in OL differentiation, NPCs were transduced with pLenti-III-miR-184/pLenti-III-miR-Off-184 or miR-184 mimics and evaluated for the expression of OL-specific markers using qRT-PCR and ICC, respectively. Before transduction, flow cytometry analysis of initial cells demonstrated that 84.4 ± 4.6% and 79.0 ± 3.7% of the cells expressed Nestin and SOX1, respectively (data not shown). To determine the efficacy of pLenti-III-miR-184 transduction, the expression level of miR-184 was examined in treatment and control groups by qRT-PCR (Fig. 1c).

OLIG2, followed by an NKX2.2 expression, has been shown to be expressed in early pre-OPCs. Therefore, OLIG2 and NKX2.2 were selected as early OPC-specific markers in this study. Moreover, MBP, which is expressed at the terminal differentiation stage of NPCs, was considered as a later-stage marker of OL differentiation. Four days after transfection with mimics, the cells were stained via stage-specific pre-OPC markers. Enforced expression of miR-184 resulted in ~ 40% increase in the number of early OLIG2-positive cells. After 3 weeks, to determine whether or not OPCs are capable of converting to oligodendrocytes, the cells were placed in a growth factor-free medium for 2 days and the oligodendrocytic index was assessed. Approximately, a 15% increase in the number of late MBP-positive cells was observed in transduced NPCs compared to the control non-transduced NPCs. Furthermore, according to the image quantification of immunostaining results using ImageJ software (NIH), statistically significant increases in expression of MBP, OLIG2, and NKX2.2 were observed in transduced NPCs compared to the control non-transduced ones (Fig. 1a). These results indicated that miR-184 overexpression stimulated the OL differentiation pathway, resulting in a more rapid expression of OL-specific markers. Western blotting analysis revealed that not only does the miR-184 overexpression increase the number of OPCs expressing early- and late-stage markers, but it also upregulates OLIG2, NKX2.2, and MBP compared to controls at the protein level, suggesting a key regulatory role of miR-184 in OL differentiation (Fig. 1b).

qRT-PCR analysis showed that OL-specific genes, namely OLIG2, NKX2.2, and MBP, were mostly upregulated in cells transduced with miR-184. However, neuron- and astrocyte-enriched genes, such as glial fibrillary acidic protein (GFAP), BCL2L1, and LINGO1, as well as the neuron markers including β-tubulin-III, SOX-1, and neurofilament medium (NFM) tended to be downregulated (Fig. 1f–h).

In order to determine whether or not overexpression of miR-184 could take over the role of the growth factors added during the oligodendrocyte differentiation stage, oligodendrocyte differentiation of miR-184-transduced NPCs was evaluated in the absence of externally supplemented cytokines and other growth factors. In contrast to the transduction of pLenti-III-empty vector, miR-184 could significantly enhance the expression of oligodendrocyte-specific key genes (Fig. 1d, e). This result suggests that not only is miR-184 essential but also sufficient at least partially, to promote the differentiation of oligodendrocytes in the absence of growth factors.

### miR-184 induces oligodendrocyte differentiation in vivo

**Fig. 2:**
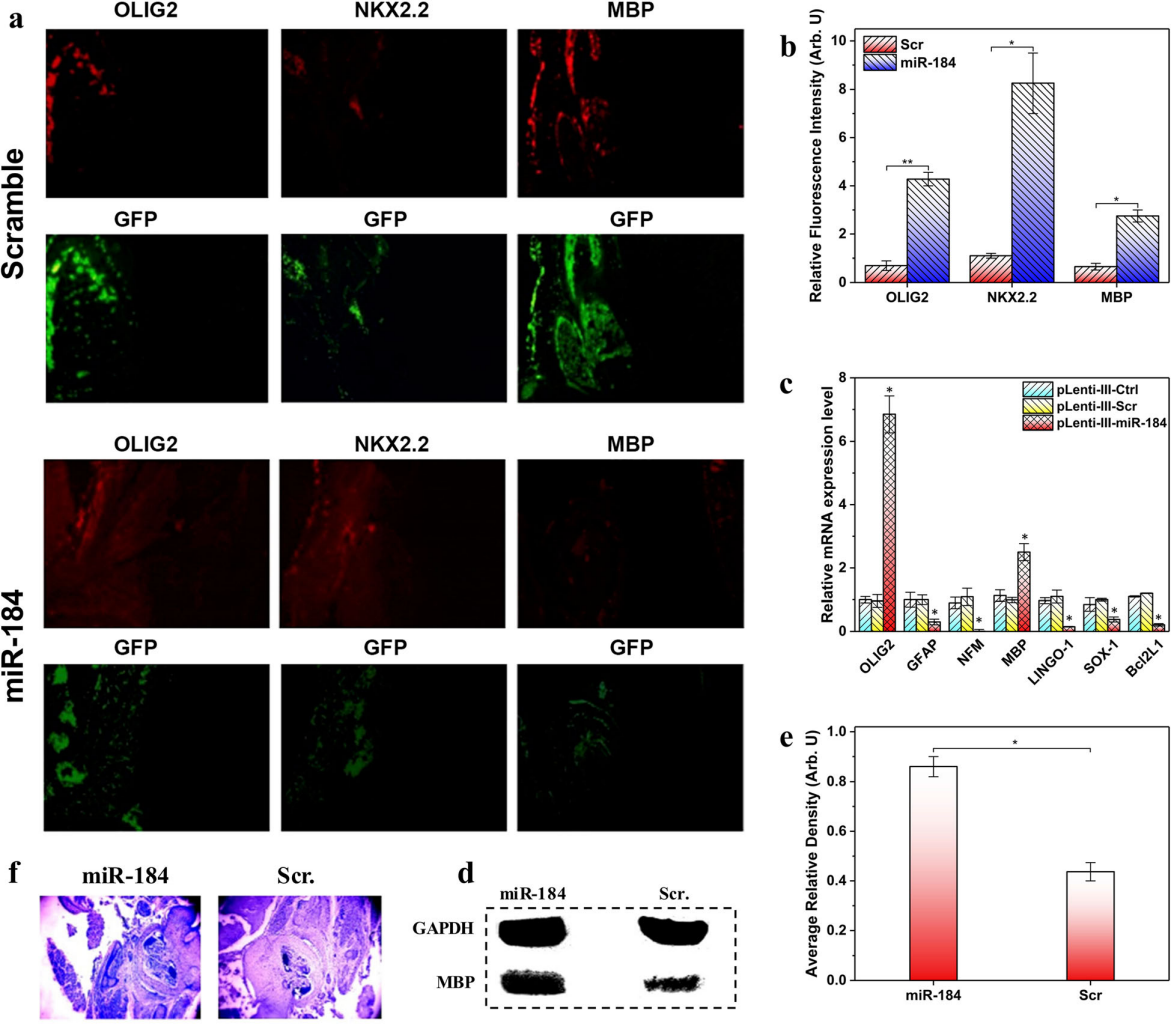
Ectopic expression of miR-184 induces OL specification in mouse cortex. **a** Mouse embryos were electroporated with pLenti-III-Ctrl or pLenti-III-miR-184 at E14.5 and harvested at E17.5. The sections of electroporated cortices were evaluated by immunostaining with antibodies against early and late OL markers, respectively. **b** Relative fluorescence intensity (G/B) representing the green fluorescence intensity (G) normalized to that of the blue one (B) was obtained for IHC results after analysis of the images by ImageJ software. Unpaired *t*-test method was employed in each case to compare the amounts of results statistically. **c** Expression of lineage-specific markers was measured on the electroporated cortices (*n* = 3) at a defined cortical area (1 mm^2^). β-Actin was used as an internal control. Electroporation of miR-184 induced an increase in the OL genes and a decrease in the astrocyte and neuron-specific markers in the cortex. Data represent mean ± SD. (*P < 0.05). **d** MBP protein level was also evaluated by western blotting in the electroporated cortices (*n* = 3) at a defined cortical area. GAPDH is used as the control in western blot analysis. **e** Average relative density of protein bands was obtained after densitometric analysis of the bands by ImageJ software followed by normalization to that of GAPDH as the internal loading control. Unpaired *t*-test method was employed to compare the amounts of results statistically. *P value < 0.05. **f** Myelination was examined using luxol fast blue-crystal violet staining on electroporated sections

To address the role of miR-184 in oligodendrocyte development and myelination in vivo, miR-184 expressing vector was electroporated into one side of the neocortical ventricular zone of developing mouse embryos at E14.5. The embryos were harvested at E17.5 before the differentiation of endogenous oligodendrocytes. IHC results demonstrated that miR-184 overexpression induced a significant increase in the expression of oligodendrocyte markers in the electroporated side of the cortex (Fig. 2a, b). Moreover, an increase in the expression level of OL-specific genes was observed by qRT-PCR, which was not the case for the neurons and astrocyte-specific genes (Fig. 2c). To assess the myelination level, myelin was histologically stained using Luxol fast blue (LFB) on collected sections. Myelin-staining was qualitatively improved in the miR-184 electroporated embryonic samples compared to the control sections (Fig. 2f). Quantification of the blue color density demonstrated that in the miR-184-electroporated embryos, myelination level was 1.54 times higher than that of the control sections. Western blot analysis shows that the MBP protein level was upregulated in mouse cortex by miR-184 overexpression compared with pLenti-III-Scr transduction (Fig. 2d, e).

Our data confirm the role of miR-184 in promoting the progression of NPCs into oligodendrocyte lineage in the developing mouse cortex.

### Knockdown of miR-184 leads to reduced OL differentiation in the central nervous system

**Fig. 3:**
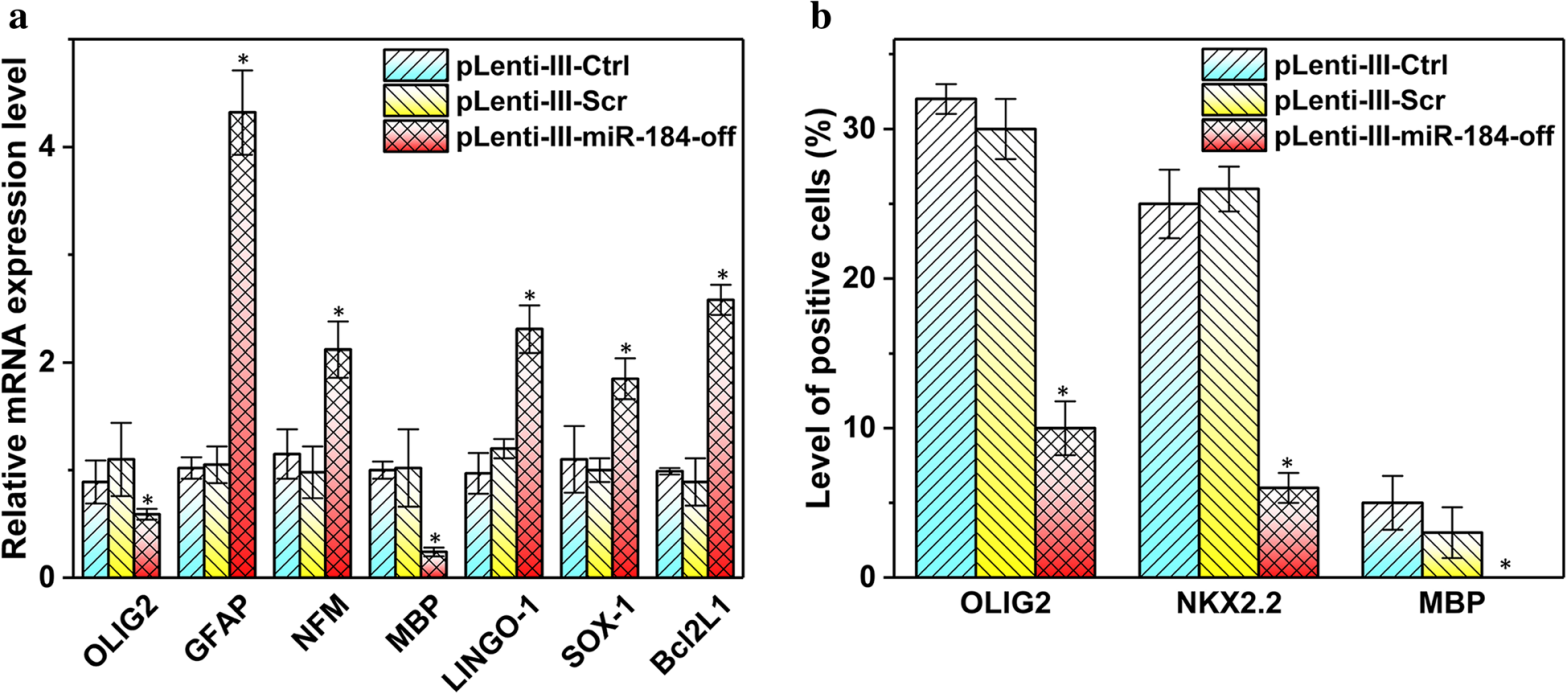
Downregulation of miR-184 by antimiR reduces the expression of OL lineage markers. **a** NPCs were transduced with pLenti-III-miR-Off-184, pLenti-III-Scr, and pLenti-III-Ctrl. mRNA levels of lineage-specific markers were quantified 7 days post-transduction. β-actin was used as an internal control. Columns, mean of three different experiments; bars, SD; (∗*P* < 0.01). **b** Histogram depicts the percentage of OLIG2+ and NKX2.2+ cells 7 days in oligo medium and MBP+ after 2 days in growth factors free oligo medium using ICC. Knockdown of miR-184 decreased the percentage of OLIG2+ and NKX2.2+ cells significantly compared to the control. Interestingly, no MBP+ cells were observed in the antimiR group. Data obtained from at least three independent experiments. In each ICC experiment, 100 cells were counted and the number of marker-positive cells was reported as a percentage. Data represent mean ± SD. (*P < 0.05)

To further understand the role of endogenous miR-184 for oligodendrocyte formation, NPCs were transduced by pLenti-III-miR-Off-184 to inhibit miR-184 activity. The gene expression analysis showed that knockdown of miR-184 significantly reduced the expression of OLIG2, NKX2.2, and MBP, while the expression of neurons and astrocyte-specific genes were greatly induced compared to control (Fig. 3a). Inhibiting miR-184 significantly reduced the percentage of early and late markers of OL lineage in transduced NPCs by ICC (Fig. 3b). These observations suggest that miR-184 knockdown blocks oligodendrocyte differentiation and maturation while promoting differentiation of neurons and astrocytes.

### miR-184 inhibits SOX1, LINGO1, and BCL2L1

**Fig. 4:**
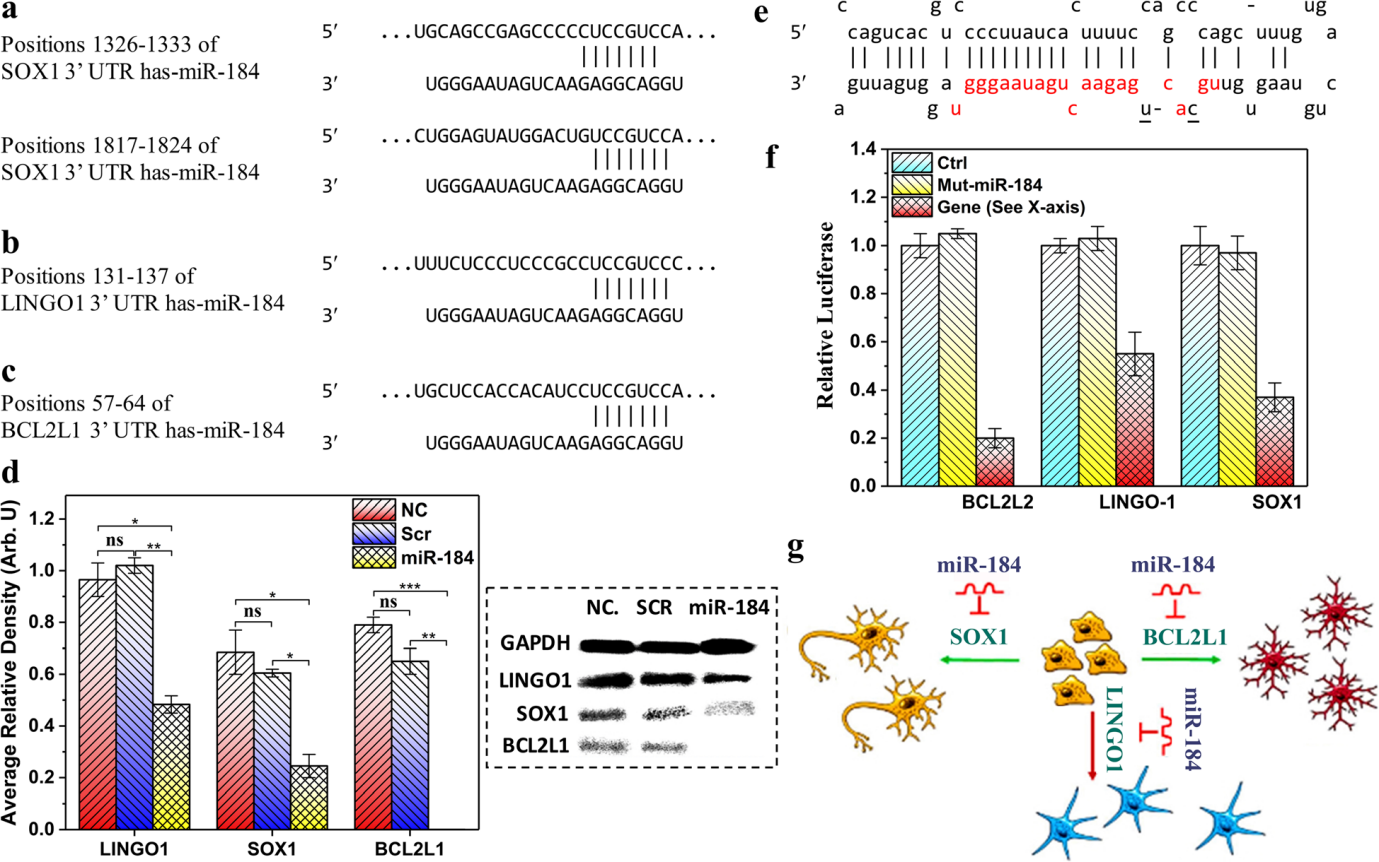
MiR-184 directly targets SOX1, LINGO1, and BCL2L1. Schematic representation of the miR-184 binding sites in the 3′-UTR of predicted target sequences: SOX1 (**a**), LINGO1 (**b**), and BCL2L1 (**c**). **d** Right: SOX1, LINGO1, and BCL2L1 proteins in NPCs were measured by western blotting 96 h post-transduction. GAPDH was used as an internal loading control. Left: Average relative density of protein bands was obtained after densitometric analysis of the bands by ImageJ software followed by normalization to that of GAPDH as the internal loading control. One-way ANOVA method was used in each case to compare the amounts of results statistically. ns: non-significant (*P* value > 0.05), *P value < 0.05, **P value < 0.01, ***P value < 0.001. **e** To construct pCDH-Mut-miR-184, two mutations were generated in the seed region of miR-184 and pre-miR-184 mutated form was cloned into pCDH-GFP. The mir-184 mature sequence is designated in red and the mutated form of the nucleotides was indicated in underlined and black. **f** HEK293 cells were co-transfected with the pLenti-III-miR-184 or pCDH-Mut-miR-184 and 3′-UTR-pSICHEK2 reporter vectors. Activities of the SOX1-, LINGO1-, and BCL2L1-bearing pSICHEK2 vectors were significantly declined in pLenti-III-miR-184 transfected cells compared to pLenti-III-Ctrl and pCDH-Mut-miR-184 vectors used as negative controls. Luciferase activity was detected 48 h after transfection. Values are means ±SD of the ratio of the luciferase activity from three independent runs (*P < 0.001). **g** Schematic model of OL differentiation pathway induced by miR-184 through Inhibition of SOX1, LINGO1, and BCL2L1. During the NPC differentiation, miR-184 suppresses the expression of neural cell fate determination and differentiation gene (SOX1) leading to the neuron generation inhibition. It also suppresses the expression of BCL2L1 to block the astrocytic differentiation at the point of astrocyte/oligodendrocyte lineage divergence. Furthermore, inhibition of LINGO1 could facilitate OL lineage differentiation. Stimulatory impact on differentiation is shown by green lines with arrowheads and inhibitory roles in differentiation are indicated by the red line with an arrowhead

To further investigate the molecular mechanisms of the miR-184 regulatory role in OL development and axon myelination, computational analysis using TargetScan, miRanda, and mirBase prediction algorithms were used to predict target genes involved in regulation by miR-184, which play roles in neurogenesis, astrocytogenesis, and oligodendrogenesis. Based on the scores, SOX1 with two recognition sites (positions 1326–1333 and 1817–1824 of the 3′-UTR), LINGO1 (position 131–137), and BCL2L1 (position 57–64) (Fig. 4a–c) were predicted to interact with miR-184.

SOX1 plays a direct role in neural cell fate determination and differentiation, and its overexpression is sufficient to induce neuronal lineage commitment [25–28]. LINGO1 is a key negative regulator of myelination that inhibits the differentiation of OPCs [29, 30]. Finally, BCL2L1 is a highly expressed gene in astrocytes [31]. Therefore, the predicted target proteins were analyzed 96 h after miR-184 transduction in NPCs. It turned out that ectopic expression of miR-184 dramatically reduced the protein levels of SOX1, LINGO1, and BCL2L1 (Fig. 4d). The 3′-UTR of all three genes was cloned into the 3′-position of the luciferase reporter gene of the pSICHEK-2 plasmid. In a parallel experiment, the conserved seed sequence of miR-184 within nucleotides 2–8 was specifically mutated. HEK293T cells were transiently co-transfected with the aforementioned 3′-UTR-reporter constructs and WT-pLenti-III-miR-184 or Mut-pCDH-miR-184 (Fig. 4e). In the presence of the WT-miR-184 expression vector, the constructs bearing the 3′-UTR of SOX1, LINGO1, and BCL2L1 genes led to a significant decrease in reporter activity compared to what was obtained for the control. As expected, the activity of the reporter construct carrying a mutated pre-miR-184 was not altered (Fig. 4f). These assays clearly demonstrated that miR-184 effectively targets SOX1, LINGO1, and BCL2L1 genes.

## Discussion

The progressive loss of CNS myelin as a consequence of oligodendrocyte injury and remyelination failure is the hallmark of some neurodegenerative autoimmune diseases, such as multiple sclerosis and leukodystrophies [32]. Remyelination is a complex biological process for the creation of a thinner than normal myelin sheath on demyelinated axons, aiming to protect the axons from further damage and overall degeneration in order to restore conductance in the CNS [33].

It is generally accepted that OPCs, rather than mature oligodendrocytes, are the main cells responsible for the remyelination of demyelinated axons. Thus, OPCs are considered as a promising cell population for MS therapeutic approaches [34–36]. However, little is known about the fundamental regulatory mechanisms that control the differentiation of progenitor cells into OPCs.

Over the past few years, several groups have investigated the roles of individual miRNAs in OL fate determination from progenitor cells. For example, miR-219 and miR-338 were identified as oligodendrocyte-specific miRNAs in the spinal cord that target negative regulators of oligodendrocyte differentiation, including transcription factors such as Sox6 and Hes5 [20]. miR-23a was also reported as another key miRNA which is approximately fivefold more expressed during OL maturation and enhances both oligodendrocyte differentiation and myelin synthesis through suppression of lamin B1 [37]. The miR-17-92 cluster has been identified to be both necessary and sufficient to enhance in vivo and in vitro OPC proliferation [38]. miR-7a has been reported to highly express in OPCs, and its ectopic expression in either NPCs or embryonic mouse cortex leads to the generation of OL lineage cells [21].

miRNAs also play essential roles in distinct stages of OL differentiation and myelination. Letzen et al. evaluated the miRNA profile in eight stages of OL differentiation starting from ES cells and indicated that miR-184 was the highest upregulated miRNA at GP to early OPC transition, becoming one of the top upregulated miRNAs throughout OPC to OL transition [22]. Thus, it is so likely that enforced expression of miR-184 in NPCs may lead to fate commitment of oligodendrocyte lineage with the expense of neurons and astrocytes.

Given all the reported information for the role of miRNAs in cell differentiation and the lack of data for miR-184, our focus turned to better understand the mechanism of miR-184 action in oligodendrocyte fate determination. To address this, miR-184 was overexpressed in the NPCs for further analysis. Immunostaining with OPC makers, i.e., OLIG2 and NKX2.2, in miR-184-transfected NPCs resulted in a significant increase in the number of OPCs, whereas blocking the endogenous activity of miR-184 led to a reduction in the number of committed cells, totally consistent with the qRT-PCR results. The number of OLIG2, NKX2.2, and MBP-positive cells in the pLenti-III-miR-Off-184 group was similar compared to that of the control. Overexpression of miR-184 in NPCs led to not only the upregulation of OL-specific genes but also a substantial downregulation of neuronal and astrocyte marker genes. This demonstrates that miR-184, aside from enhancing OL lineage progression, inhibits the expression of other lineage-specific genes which can potentially limit the promotion of OL differentiation from progenitor cells.

Since miR-184 can express early OL gene expression, we also hypothesize that its expression in NPCs can accelerate the OL developmental stage.

Consistent with our findings, results from the overexpression of miR-184 in the forebrain ventricle of developing embryonic mouse showed that miR-184 could be used as an oligodendrocyte specification inducer. IHC and qRT-PCR analyses all reveal a distinguished role for miR-184 in the in vivo oligodendrocyte development within the brain.

Our data show that ectopic expression of miR-184 in NPCs can allow for stimulating OPC gene expression and inducing oligodendrocyte differentiation in the absence of multiple cytokines and growth factors that are routinely used for this purpose that further emphasizes the importance of miR-184 in OL fate determination.

miRNAs, on the other hand, are capable of regulating differentiation through complex processes including targeting several mRNA molecules in multiple dependent and independent pathways. To understand molecular mechanisms by which miR-184 regulates OL lineage commitment, we looked to find which mRNA target of miR-184 would be involved in neuronal, astrocyte, and OL differentiation. Using the TargetScan algorithm [39], we predicted several targets with a clear functional role in differentiation of NPCs including SOX1, BCL2L1, and LINGO1, and the predicted targets were then validated using luciferase assays. SOX1, BCL2L1, and LINGO1 were identified to be bona fide targets of miR-184 as their expression levels were directly controlled by miR-184 through their 3′-UTR.

Kan et al. demonstrated that SOX1 expression may promote neuronal lineage commitment through multiple pathways including direct binding to the Hes1 promoter resulting in Notch signaling attenuation via suppressing Hes1 transcription; binding to h-catenin and suppression of h-catenin-mediated TCF/LEF signaling leading to attenuating the wnt signaling pathway; and promoting the existence of cells from cell cycle and upregulating transcription of the proneural bHLH transcription factor neurogenin1 [25]. Thus, SOX1 signaling appears to play pivotal roles in neural cell fate determination and differentiation [26–28]. Our study showed that miR-184, through targeting SOX1, can act as a barrier against neuronal differentiation paving the path for OL differentiation.

BCL2L1 is another target that was found by our computational studies which get co-expressed with GFAP in various samples of astrocyte tissue and plays a strong role in astrocyte function [31]. Latzen et al. proposed that via binding to BCL2L1, miR-184 may prevent astrocytic differentiation at the point of astrocyte/oligodendrocyte lineage divergence. In the current study, we experimentally proved by luciferase assay that BCL2L1 is a direct target of miR-184 [22]. Hence, downregulation of BCL2L1 could emerge as the second effector through which miR-184 leads to OL commitment instead of astrocyte lineage.

LINGO1 was the next target that was identified by computational studies which is a key negative regulator of myelination as well as a CNS-specific membrane protein in neurons. LINGO1, along with Nogo receptor 1 (NgR1) and p75/tumor necrosis factor orphan receptor (TROY), organizes the myelin inhibitor receptor complex involved in blocking axonal regeneration [29, 30]. In oligodendrocytes, LINGO1 plays key negative regulatory roles in oligodendrocyte differentiation and myelination process via inhibiting ErbB2 translocation and activation in lipid rafts [40]. Here, we present experimental evidence that miR-184 directly targets LINGO1 and subsequently facilitates OPC differentiation and myelination.

## Conclusions

In conclusion, the effects of miR-184, at least in part, can be explained by targeting SOX1 and BCL2L1, which in turn inhibit neuron differentiation (Fig. 4g). This can reduce astrocyte differentiation and LINGO1 level leading to OL differentiation and myelination. Together, we provide evidence that miR-184 is an important regulator of oligodendrocyte development and repression of its targets could be one of the critical steps necessary for driving NPCs to OPCs and terminal differentiation of oligodendrocytes. Our study introduces miR-184 as an oligodendrocyte-specific miRNA in CNS whose overexpression is sufficient to promote OPC differentiation even in the absence of differential growth factors, providing a resource for future studies on miRNA actions combined with transcriptional regulators in oligodendrocytes. In the present study, we used a lentiviral vector for NPC transduction. However, for cell therapy and clinical application, adenoviral vectors could be the most attractive delivery tools due to the relative ease of manipulation and high vector titers without integrating its cargo into the host genome. Furthermore, given that many patients do not respond optimally to immunomodulatory drugs, the results of this study can be used in combined therapy studies with standard MS drugs and may offer new therapeutic options for treating patients with MS.

## Additional file

Additional file 1: **Table S1.** Primers for 3′UTR cloning. (DOCX 12 kb)

Additional file 2: **Table S2.** Primers for qRT-PCR of genes and miRNAs. (DOCX 13 kb)

## Abbreviations

3′-UTR: 3′-Untranslated region
BCL2: B cell lymphoma 2
BCL2L1: BCL2 Like 1
bFGF: Basic fibroblast growth factor
bHLH: Basic helix-loop-helix
CNS: Central nervous system
DMEM: Dulbecco’s modified Eagle’s medium
ErbB2: Erb-B2 receptor tyrosine kinase 2
FBS: Fetal bovine serum
GFAP: Glial fibrillary acidic protein
GFP: Green fluorescent protein
GP: Glial-restricted precursor
HEK293T: Human embryonic kidney cells
HES1: Hairy and enhancer of split-1
hiPSCs: Human-induced pluripotent stem cells
ICC: Immunocytochemistry
IFN-β: Interferon-beta
IHC: Immunohistochemistry
LFB: Luxol fast blue
LINGO1: Leucine rich repeat and Immunoglobin-like domain-containing protein 1
MBP: Myelin basic protein
miRNAs: MicroRNAs
MS: Multiple sclerosis
NgR1: Nogo receptor 1
NKX2.2: NK2 homeobox 2
NPCs: Neural progenitor cells
OL: Oligodendrocyte
OPCs: Oligodendrocyte progenitor cells
PDGF: Platelet-derived growth factor
PDL: Poly-d-lysine
qRT-PCR: Quantitative reverse transcription polymerase chain reaction
SD: Standard deviation
SOX1: SRY-box 1
SRY: Sex-determining region Y
TCF/LEF: T cell factor/lymphoid enhancer factor signaling
TROY: Tumor necrosis factor orphan receptor
