# Supplementary material for: A critical role for miR-184 in the fate determination of oligodendrocytes
Source: Stem Cell Res Ther. 2019 Mar 29;10:112. doi: 10.1186/s13287-019-1208-y (PMC6440085; doi:10.1186/s13287-019-1208-y)
Supplement: Supplementary file 1 — Table S1. Primers for 3′UTR cloning. (DOCX 12 kb) [file 13287_2019_1208_MOESM1_ESM.docx]

Table S1. Primers for 3′UTR cloning

| **Genes** | **Primer sequences** |
| --- | --- |
| **BCL2L1** | FW, 5′-CGCTCGAGGGAACTCTATGGGAACAATGC-3′;  RW, 5′-AAGCGGCCGCTCTAGCCAGTCCAGAGGTGAG-3 |
| **LINGO-1** | FW, 5′-GCGCTCGAGCGACATCAAGACCCTCATCAT -3′;  RW, 5′-AAGCGGCCGCTCTCTCCACTGACTGTGCTGTC-3 |
| **SOX1** | FW, 5′-GCGCTCGAGAGGCAGGTCCAAGCACTTAC-3′;  RW, 5′-AAGCGGCCGCCTAAGTCGTAGTGGTGCTCAGC -3 |
