# Supplementary material for: A critical role for miR-184 in the fate determination of oligodendrocytes
Source: Stem Cell Res Ther. 2019 Mar 29;10:112. doi: 10.1186/s13287-019-1208-y (PMC6440085; doi:10.1186/s13287-019-1208-y)
Supplement: Supplementary file 2 — Table S2. Primers for qRT-PCR of genes and miRNAs. (DOCX 13 kb) [file 13287_2019_1208_MOESM2_ESM.docx]

Table S2. Primers for qRT-PCR of genes and miRNAs

| **Genes** | **Primer sequences** |
| --- | --- |
| **OLIG2** | FW, 5′-GCT GTGAAACACTTTGGGT -3′;  RW, 5′-AAGGGTGTTACACGGCAGAC-3′ |
| **GFAP** | FW, 5′-ACCAGGACCTGCTCAATGTC-3′;  RW, 5′-ATCTCCACGGTCTTCACCAC -3′ |
| **NFM** | FW, 5′-TCAACGTCAAGATGGCTCTG-3′;  RW, 5′-TGTGTTGGACCTTAAGCTTGG-3′ |
| **MBP** | FW, 5′-ACCCCGTAGTCCACTTCTTC -3′;  RW, 5′-ACTCCCTTGAATCCCTTGTG-3′ |
| **LINGO-1** | FW, 5′-GGACATCAGCGAGAACAAG-3′;  RW, 5′-TCAGGTTGCATTTCTCCAG-3′ |
| **SOX-1** | FW, 5′-TGACGCGTGTCCCCCACTCA-3′;  RW, 5′-ACGCGGAGGAAAGTGAGCGC -3′ |
| **Bcl2L1** | FW, 5′-TAAGGCGGATTTGAATCTC-3′;  RW, 5′-ATAATAGGGATGGGCTCAAC-3′ |
| **HPRT** | FW, 5′-CCT GGC GTC GTG ATT AGT G -3′;  RW, 5′-TCA GTC CTG TCC ATA ATT AGT CC-3′ |
| **MiR-184** | FW, 5′-ATCTTTTTGCGGTCTGG -3′;  RW, 5′-GAGCAGGGTCCGAGGT |
| **SNORD47** | FW, 5′-ATCACTGTAAAACCGTTCCA-3′;  RW, 5′-GAGCAGGGTCCGAGGT -3′ |
